# Diagnostic odyssey of acute disseminated encephalomyelitis in children

**DOI:** 10.1038/s41598-021-01519-5

**Published:** 2021-11-09

**Authors:** Yoko Takahashi, Itaru Hayakawa, Yuichi Abe

**Affiliations:** 1 Division of Neurology, National Center for Child Health and Development, 2-10-1, Okura, Setagaya-ku, Tokyo, Postal Code 157-8535 Japan; 2 Center for Postgraduate Education and Training, National Center for Child Health and Development, Tokyo, Japan; 3 Department of Pediatrics, Tohoku University School of Medicine, Sendai, Japan

**Keywords:** Paediatric research, Demyelinating diseases

## Abstract

We aimed to determine whether acute disseminated encephalomyelitis (ADEM) diagnosis in children is delayed, and if so, to identify the clinical risk factors of delayed diagnosis. Standardised data were collected from children with ADEM from 2003 to 2020. Overall diagnostic delay (time between symptom onset and ADEM diagnosis), physicians’ delay (between the first medical visit and ADEM diagnosis), and patients’ delay (between symptom onset and the first medical visit) were analysed. Thirty ADEM patients were identified, including 16 (54%) with neurological deficits at discharge. Overall, physicians’, and patients’ delays were 9 (interquartile range [IQR] 6–20.5), 5.5 (IQR 3–14), and 4 (IQR 2–8) days, respectively. Overall delay was significantly associated with physicians’ delay, but not with patients’ delay. There were 61 misdiagnoses among 25 (83%) patients, while 5 (17%) were diagnosed correctly at the first visit. The misdiagnoses of common respiratory and gastrointestinal infection and aseptic meningitis were associated with overall and/or physicians’ delay. Later onset of specific neurological features suggestive of ADEM was associated with all three diagnostic delays. A unique diagnostic odyssey exists in ADEM. Several clinical risk factors were associated with the diagnostic delay.

## Introduction

Acute disseminated encephalomyelitis (ADEM) is an acquired demyelinating disorder of the central nervous system (CNS) in children and young adults^1^. It typically affects the subcortical white matter and is characterised by monophasic encephalopathy and polyfocal neurological symptoms^2^. While pathophysiology, treatment, and prognosis of ADEM have been described previously^3^, ADEM diagnosis is still a clinical one since no specific serological biomarkers are available to date and brain imaging and particular antibodies are ancillary. An ADEM diagnosis is frequently challenging because the initial prodromal presentation is highly variable and non-specific, including symptoms such as fever, headache, and nausea. Furthermore, encephalopathy, the key to diagnosis based on International Pediatric Multiple Sclerosis Study Group criteria^4^, may present with vague, subtle, and transient sleepiness or irritability, especially in paediatric patients. Thus, the diagnosis of ADEM requires a high index of clinical suspicion and timely performance of diagnostic tests^4^.

Timely diagnosis is vital for early intervention and favourable prognosis in ADEM^4,5^. The mortality rate of ADEM is 1–3%, and long-term cognitive or neurological deficits affect up to 50% of paediatric patients^2,4,6,7^. A longer diagnostic odyssey leads to unnecessary medical interventions and additional costs. For example, children with ADEM may initially be treated with broad-spectrum antibiotics and antiviral agents^2^, since clinical features of ADEM often resemble those of acute CNS infections, which may lead to drug-related adverse effects and additional costs. While reducing the number of days spent due to incorrect diagnoses is unequivocally important, only a few studies have addressed diagnostic errors in ADEM to date.

In this study, we aimed to determine whether acute disseminated encephalomyelitis (ADEM) diagnosis in children is delayed, and if so, to identify the clinical risk factors of delayed diagnosis. We employed the diagnostic odyssey plot to elucidate the diagnostic process in each patient with ADEM. Our data and visual representation of the diagnostic timeline will help future physicians diagnose ADEM early in the disease course.

## Methods

### Study design and setting

This was a single-centre cohort study conducted at a national children’s medical centre from March 2003 to July 2020. The medical centre was a tertiary care referral centre in an urban area of Japan equipped with 530 ward beds, 20 paediatric intensive care unit (ICU) beds, and an emergency department with some 30,000 annual visits. We collected standardised information^2–4^ using manual chart reviews.

This study was conducted in accordance with the ethical standards of the institutional and/or national research committee and with the 1964 Helsinki declaration and its later amendments, or comparable ethical standards. The study was approved by the ethics committee/institutional review board of National Centre for Child Health and Development (number 2020–188), and written informed consent was waived due to the retrospective nature of this study by the ethics committee/institutional review board.

### Study population

Consecutive patients diagnosed with ADEM aged < 18 years old who were admitted to the centre were included. Patients were excluded if they were initially diagnosed and received treatment at other hospitals, and if they were diagnosed as multiphasic disseminated encephalomyelitis, multiple sclerosis, and neuromyelitis optica spectrum disorder during follow-up. All patients were followed up after discharge at our affiliated outpatient clinic by paediatric neurologists.

### Measures

ADEM was ascertained according to current international consensus guidelines^3^. Information on patient demographics, clinical symptoms, and disease course was collected from outpatient and inpatient charts entered by senior emergency medicine physicians, paediatric residents and attendings, and paediatric neurologists. Patient symptoms were recorded through manual chart review based on international consensus and previous reports^2–4^. Briefly, encephalopathy was defined by the international consensus as an alteration in consciousness (e.g., stupor, lethargy), or a behavioural change unexplained by fever, systemic illness, or postictal symptoms^3^. Neurological findings recorded by paediatric neurologists were retrieved. Disease onset was defined retrospectively as the date on which the first symptoms of ADEM appeared. The day of the first visit was when the patient first visited any medical services including primary care physicians, outpatient clinics, or the emergency department. Preceding infections and immunisations were defined as events within one month prior to disease onset. Incorrect diagnoses were recorded from the patient’s history when primary care physicians made the diagnoses and from chart reviews when the in-house physicians made the diagnoses.

### Analyses of the time of diagnosis

**Figure 1:**
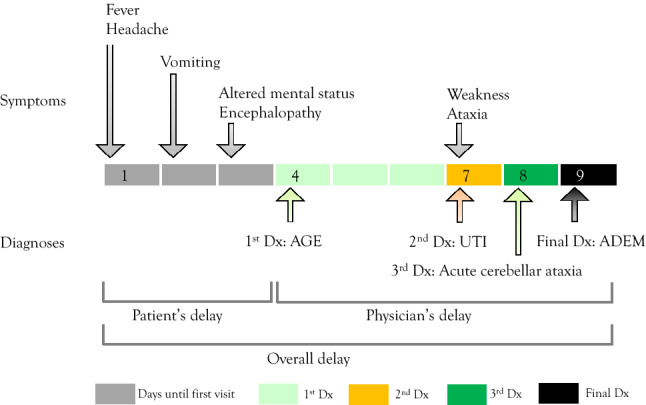
Example of the diagnostic odyssey plot. Patient #18 visited the physician on the fourth day after symptom onset. There were three misdiagnoses until the correct diagnosis of ADEM. Patient and physician delays were 3 and 6 days, respectively. Grey: days from symptom onset to first visit (patient delay); light green: days with the first diagnosis; orange: days with the second diagnosis; green: days with the third diagnosis; and black: day of final diagnosis (ADEM diagnosis). *ADEM* acute disseminated encephalomyelitis, *AGE* acute gastroenteritis, *UTI* urinary tract infection, *Dx* diagnosis.

**Figure 2:**
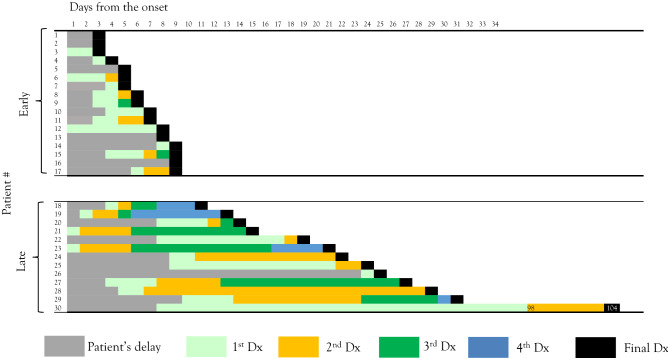
Overall diagnostic delay in paediatric ADEM. Each column shows the diagnostic odyssey plot for each ADEM case. Cases are placed in ascending order of their overall diagnostic delay. Early-diagnosed cases are those diagnosed ≤ 9 days after symptom onset. *ADEM* acute disseminated encephalomyelitis.

In order to improve visibility and aid in understanding the disease course, we graphically represented the time course for each patient into a spreadsheet, which was named the diagnostic odyssey plot (Figs. 1 and 2). Each patient with ADEM typically recognised symptoms and visited a physician, and after a few misdiagnoses, a correct diagnosis of ADEM was made (Fig. 1). Overall diagnostic delay was the interval between the onset of symptoms (first grey cell) and actual diagnosis of ADEM (black cell). Similarly, patients’ delay was the period from the onset of symptoms to the first medical visit. Physicians’ delay was the interval between the patient’s first medical visit and the day of ADEM diagnosis.

**Figure 3:**
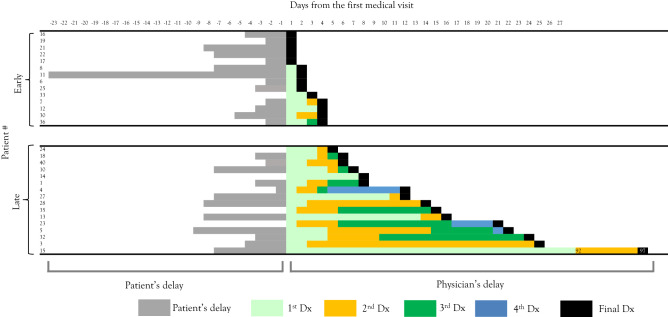
Physicians’ diagnostic delay in paediatric ADEM. Each column shows the diagnostic odyssey plot for each ADEM case. Cases are placed in ascending order of their physician’s diagnostic delay. Physicians’ early-diagnosed cases are those diagnosed ≤ 4 days after initial visit. *ADEM* acute disseminated encephalomyelitis.

**Figure 4:**
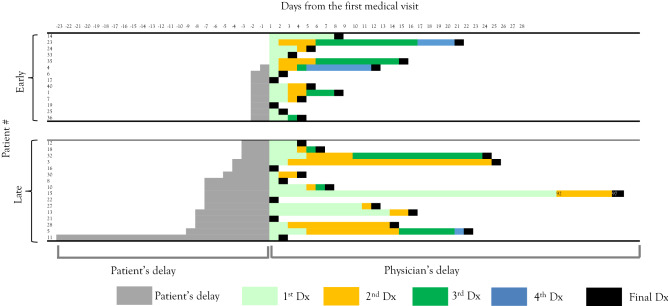
Patient attendance and diagnostic delay in paediatric ADEM. Each column shows the diagnostic odyssey plot of each ADEM case. Cases are placed in ascending order of their patient attendance delay. Patient early-attended cases are those attended ≤ 4 days after symptom onset. *ADEM* acute disseminated encephalomyelitis.

Clinical risk factors for diagnostic delay were searched by comparison between overall delayed (> 9 days after symptom onset) and early (≤ 9 days) diagnoses (Fig. 2, Tables 1 and 2); physicians’ delayed (> 4 days after initial visit) and early (≤ 4 days) diagnoses (Fig. 3, Tables 3 and 4); and patients’ delayed (> 4 days after symptom onset) and early (≤ 4 days) attendances (Fig. 4, Tables 5 and 6).

**Table 1:** Clinical parameters of the overall early-diagnosed and late-diagnosed groups.

	Total (n = 30)	Early-diagnosed group (n = 17)	Late-diagnosed group (n = 13)	*P* value
Sex (male)	16	8	8	0.48
Age at onset	5.5 (4.3–7.5)	5.5 (2.5–6.8)	6.1 (4.8–7.8)	0.5
Overall delay (days from onset to final diagnosis)	9 (6–20.5)	6 (5–8)	22 (15–27)	NA
Physicians’ delay (days from first visit to final diagnosis)	5.5 (3–14)	3 (2–4)	15 (12–22)	0.000046
Patients’ delay (days from onset to first visit)	4 (2–8)	3 (2–5)	8 (3–9)	0.087
Number of misdiagnoses	2 (1–3)	1 (1–2)	3 (2–4)	0.0002
**Symptoms**				
Fever	25	12	13	0.05
Days the symptom appears	1 (1–1)	1 (1–2.5)	1 (1–1)	0.46
Headache	24	12	12	0.19
Days the symptom appears	3 (1–4)	2.5 (1.8–3)	3 (1–5)	0.53
Encephalopathy	21	13	8	0.44
Days the symptom appears	5 (3–8)	4 (3–6)	13 (7.5–22)	0.009
Weakness	14	8	6	1
Days the symptom appears	6.5 (3.5–13)	4 (1.8–6.3)	16.5 (9–22)	0.013
Sensory deficit	17	8	9	0.28
Days the symptom appears	4 (4–11)	3.5 (2.8–4)	11 (6–14)	0.0025
Autonomic dysfunction	10	8	2	0.11
Days the symptom appears	4.5 (3- 8.8)	3.5 (2.8–5.8)	15.5 (14–17)	0.036
**Neurological exams**				
Neck stiffness	21	10	11	0.22
Paralysis	12	10	2	0.025
Autonomic involvement	9	8	1	0.04
**Laboratory data**				
White blood cells (× 10^3^/µL)	13.03 (8.56–17.31)	9.48 (6.54–14.15)	15.04 (12.36–22.26)	< 0.01
Stab (%)	74.5 (63–81)	68 (50–74)	81 (78–84)	0.00037
C-reactive protein (mg/dL)	0.45 (0.2–1.4)	0.2 (0.2–0.7)	1.4 (0.9–2.8)	0.0058
**CSF**				
Days the CSF were collected	8 (4–9)	5 (3–8)	11 (12–22)	0.0042
CSF-white cell count (/µL)	116 (30–330)	82	164	> 0.05
CSF-stab (/µL)	13 (4–62)	6	28	0.06
**Interventions**				
ICU admission	12	10	2	0.025
Broad-spectrum antibiotics	12	5	7	0.26
**Prognosis**				
Death	0	0	0	NA
Initial EDSS at admission	8 (5–9)	8.5 (8–9)	5 (3–7.5)	0.0035
Neurological deficits at discharge	16	14	2	0.0006

Numbers in parentheses denote interquartile ranges.

*CSF* cerebrospinal fluids, *ICU* intensive care unit, *EDSS* expanded disability status score, *NA* not applicable.

**Table 2:** Frequency of common misdiagnoses in the overall early-diagnosed and late-diagnosed groups.

	Early-diagnosed group (n = 17)	Late-diagnosed group (n = 13)	*P* value
Common respiratory infection	1	8	0.0016
Bacterial meningitis	3	6	0.12
Aseptic meningitis other than HSV	2	7	0.02
HSV encephalitis	3	2	1
Common gastrointestinal infection	3	2	1
Fever of unknown origin	0	3	0.07
Others	12	9	–
Total	24	37	–

*HSV* herpes simplex virus.

**Table 3:** Clinical parameters of the physician early-diagnosed and late-diagnosed groups.

	Physicians’ early-diagnosed group (n = 14)	Physicians’ late-diagnosed group (n = 16)	*P* value
Physicians’ delay (days from first visit to final diagnosis)	2.5 (1–3.8)	13 (7.8–21)	NA
Numbers of misdiagnosis	1 (1–2)	3 (2–4)	0.00015
**Symptoms**			
Weakness	6	8	0.73
Day the symptom appears	2.5 (1.3–4.5)	11 (6.8–19.3)	0.011
Sensory deficit	7	10	0.71
Day the symptom appears	3 (2.5–4)	9 (4.5–13.5)	0.0041
Autonomic dysfunction	6	4	0.44
Day the symptom appears	3 (2.3–6.8)	8.5 (4.8–14)	0.086
**Laboratory data**			
White blood cells (× 10^3^/µL)	9.08 (5.722–13.962)	15.735 (12.015–19.575)	< 0.001
Stab (%)	67 (45–75)	80 (73–84)	0.0043
**CSF**			
Days the CSF collected	5 (3.3–8)	8 (5.8–14)	0.078
CSF-WCC (/µL)	74.5 (19–158)	157 (74–527)	> 0.05
CSF-stab (/µl)	4.5 (2.3–16)	22 (10–115)	0.024
**Prognosis**			
Neurological deficits at discharge	8	5	0.26

Numbers in parentheses denote interquartile ranges.

*CSF* cerebrospinal fluids, *ICU* intensive care unit, *EDSS* expanded disability status score, *NA* not applicable.

**Table 4:** Frequency of common misdiagnoses in the physician early-diagnosed and late-diagnosed groups.

	Physician early-diagnosed group (n = 14)	Physician late-diagnosed group (n = 16)	*P* value
Common respiratory infection	0	9	0.00090
Bacterial meningitis	2	7	0.12
Aseptic meningitis other than HSV	2	7	0.12
HSV encephalitis	3	2	0.64
Common gastrointestinal infection	0	5	0.045
Fever of unknown origin	0	3	0.23
Others	10	11	–
Total	17	44	–

*HSV* herpes simplex virus.

**Table 5:** Clinical parameters of the patient early-attended and late-attended groups.

	Patient early-attended group (n = 14)	Patient late-attended group (n = 16)	*P* value
Physicians’ delay (days from first visit to final diagnosis)	4.5 (3–8.8)	6.5 (2–17.5)	0.54
Number of misdiagnoses	2 (1–3)	2 (1–3)	0.91
**Symptoms**			
Fever	9	16	0.01
Day the symptom appears	1 (1–1)	1 (1–1.8)	0.65
Seizure	6	5	0.7
Day the symptom appears	2 (2–2.5)	6 (5–9)	0.0048
Vomit	9	11	1
Day the symptom appears	1 (1–1)	3 (2.5–6.5)	0.011
Encephalopathy	8	13	0.23
Day the symptom appears	3 (1.8–3.3)	8 (6–17)	0.00068
Sensory deficit	10	7	0.2
Day the symptom appears	4 (3–4)	11 (6–18)	0.0075
**Neurological exams**			
Neck stiffness	11	10	0.39
Autonomic involvement	7	2	0.04
**Laboratory data**			
White blood cells (× 10^3^/µL)	13.355 (9.855–16.627)	12.88 (7.555–17.3)	0.45
Stab (%)	75 (68–78)	75 (52–82)	0.98
**CSF**			
Days the CSF collected	4 (3–5)	9 (8–16)	
CSF-WCC (/µL)	102 (38–331)	116 (36–265)	> 0.10
CSF-stab (/µL)	18 (4–62)	10 (4.8–40)	0.85

Numbers in parentheses denote interquartile ranges.

*CSF* cerebrospinal fluids, *ICU* intensive care unit, *EDSS* expanded disability status score, *NA* not applicable.

**Table 6:** Frequency of common misdiagnoses in the patient early-attended and late-attended groups.

	Patient early-attended group (n = 14)	Patient late-attended group (n = 16)	*P* value
Common respiratory infection	4	5	1
Bacterial meningitis	4	5	1
Aseptic meningitis other than HSV	5	4	0.69
HSV encephalitis	3	2	0.64
Common gastrointestinal infection	3	2	0.64
Fever of unknown origin	1	2	1
Others	11	10	–
Total	31	30	–

*HSV* herpes simplex virus.

### Statistical analyses

Data were analysed using SPSS 24.0 statistical software (IBM, Armonk, NY, United States). Demographic and clinical characteristics of the study group (n = 30) were analysed using descriptive statistics. Comparisons between early- and late-diagnosed groups were made using a nonparametric two-sided Mann–Whitney U test for continuous variables and Fisher's exact test for categorical variables. Statistical significance was set at *P* < 0.05.

## Results

### Cohort characteristics

Thirty-four children were diagnosed with ADEM during the study period. Based on the eligibility criteria, four patients were excluded and 30 were included in final analyses (Table 1). The median age was 5.5 years (interquartile range [IQR], 4.3–7.5 years). Boys comprised 53% (16/30) of the population. Fever (25/30), headache (24/30), encephalopathy (21/30), and neck stiffness (21/30) were common. Preceding infections (11/30) or immunizations (8/30) were also common. Common neurological abnormalities were rigid neck (23/30) and exaggerated deep tendon reflex (21/30). These frequencies were comparable to those reported in the previous literature^2^. The median hospital admission period was 21 days (IQR, 16–42 days). Intensive care unit (ICU) admission occurred in 12/30 patients.

### Overall diagnostic delay

The overall diagnostic delay was 9 days (IQR, 6–20.5 days) (Table 1). Seventeen patients (57%) were diagnosed within 9 days from the onset of symptoms and constituted the early-diagnosed group. The remaining 13 patients (43%) constituted the late-diagnosed group (Fig. 2).

Four clinical risk factors of overall diagnostic delay were found. First, the late-diagnosed group was more frequently misdiagnosed with common respiratory infection (*P* = 0.0016) and aseptic meningitis other than herpes simplex virus (HSV) (*P* = 0.02) than the early-diagnosed group (Table 2). Second, later onset of specific neurological features suggestive of ADEM was associated with diagnostic delays. For example, encephalopathy, weakness, sensory deficit, and autonomic dysfunction developed significantly later during the disease course in the late-diagnosed group than in the early-diagnosed group (Table 1). In contrast, fever and headache developed early during the disease course equally in the two groups. Third, elevated white blood cells in complete blood count and C-reactive protein were associated with delayed diagnosis (Table 1). While increased inflammatory markers suggest severe disease, these markers might have caused the ascertainment bias to the treating physicians. Fourth, less severe disease was associated with overall diagnostic delay. This was characterised by lower expanded disability status scores at admission (*P* = 0.0035), less frequent ICU admission (*P* = 0.025), and less frequent neurological deficits at discharge (*P* = 0.0006) in the late-diagnosed group than in the early-diagnosed group.

### Clinical risk factors of diagnostic delay related to physicians’ delay

Next, we investigated the clinical risk factors associated with a diagnostic delay attributable to physicians’ delay. The diagnostic odyssey plot presented in Fig. 2 was reformatted by filtering all cases by duration from the first hospital visit to the final diagnosis of ADEM, and was dichotomised as physicians’ early-diagnosed group versus physicians’ late-diagnosed group (Fig. 3). Only five patients were diagnosed correctly with ADEM at the first encounter. The median delay from the first visit to the diagnosis of ADEM was 2.5 days (IQR, 1–3.8 days) in the early-diagnosed group and 13 days (IQR, 7.8–21 days) in the late-diagnosed group (Table 3). Neurological deficits at discharge were comparable between the two groups (8/14 in the early-diagnosed group versus 5/16 in the late-diagnosed group, *P* = 0.26) (Table 3).

Two clinical risk factors of physicians’ diagnostic delay were identified. First, later onset of specific neurological features suggestive of ADEM, such as weakness (*P* = 0.011) and sensory deficit (*P* = 0.0041), was associated with physicians’ diagnostic delays (Table 3). Second, misdiagnoses of common respiratory and gastrointestinal tract infections led to physicians’ diagnostic delay (Table 4).

### Diagnostic delay related to patients’ delay

Next, we investigated the diagnostic delay attributable to patients’ delay. The diagnostic odyssey plot in Fig. 2 was reformatted by filtering all cases by duration from the symptom onset to the first medical visit (Fig. 4). Interestingly, physicians’ diagnostic delay did not significantly differ between patients’ early-attended group and late-attended group (*P* = 0.54, Table 5). This trend indicates that patients’ behavioural characteristics in seeking medical attention were not a contributing factor to the physicians’ delay in ADEM. Indeed, the total number of misdiagnoses did not differ between the patients’ early-attended and late-attended groups, and the types of common misdiagnoses were similar between the two groups (Table 6).

Although patients’ delay did not contribute to the overall diagnostic delay (Table 1), we found two clinical risk factors of patients’ late attendance. First, later onset of specific neurological features suggestive of ADEM, such as seizure (*P* = 0.0048), encephalopathy (*P* = 0.00068), and sensory deficit (*P* = 0.0075), was associated with patients’ late attendances. Second, autonomic involvement was common in the patients’ late attended group (*P* = 0.04).

## Discussion

In this study, we explored diagnostic delays and the unique profiles of the diagnostic odyssey in children with ADEM. We showed that 9 days elapsed between symptom onset to diagnosis of paediatric ADEM, and there were numerous misdiagnoses before the final diagnosis. We have identified clinical risk factors for the overall late-diagnosed group, physicians’ late-diagnosed group, and patients’ late-attended group. This study will help future physicians diagnose ADEM early in the disease course. We also provided a list of misdiagnoses in paediatric ADEM (Table 2). The common misdiagnoses were both neurologic (aseptic meningitis, bacterial meningitis, and HSV encephalitis) and non-neurologic (common respiratory infection, common gastrointestinal infection, and fever of unknown origin).

### Diagnostic odyssey plot

“Diagnostic odyssey” is a term coined by rare disease researchers that underscores the suffering of patients with rare diseases and their families in a long sequence of investigations and referrals^8–14^. Patients with rare diseases tend to suffer from misdiagnoses and associated unnecessary adverse effects to their physical, psychological, and social wellness^8^. To our knowledge, there have been no educational materials for unexperienced learners to observe the diagnostic odyssey of a certain rare disease in a manner that is fair and graphically easy to understand. To end the odyssey, one must first understand the journey^14^. Herein, we developed a diagnostic odyssey plot (Figs. 1 and 2) and applied it to ADEM. The plot provided a clear and easy strategy to grasp “at-a-glance” overview of this rare disease, including visual information on the overall, physician-derived, and patient derived diagnostic delays; the length and frequency of each misdiagnosis; and the time spent without diagnoses. By reformatting the plot as a spreadsheet, one can analyse the overall, physician-derived, and patient-derived diagnostic delays of the cohort. This plot is simple to create and can be applied to other rare and often misdiagnosed diseases to understand their unique diagnostic odyssey patterns. Establishing structural diagnostic processes and identifying patterns of misdiagnosis using the diagnostic odyssey plot may help physicians refine their clinical decision-making skills, thereby improving patient outcomes through early diagnosis and timely treatment interventions, and avoiding unnecessary treatment.

### Clinical risk factors of delayed diagnosis of ADEM

Early diagnosis and prompt treatment of ADEM has led to improvements in neurological and cognitive outcomes, as well as the avoidance of unnecessary antibiotic and anti-viral drug administration^15,16^. High-dose methylprednisolone is the first-line treatment for ADEM and prevents further progression, leading to a favourable prognosis^17^. Since ADEM lacks any specific serological biomarkers, its diagnosis depends on the clinicians’ suspicion through medical history collection and neurological examinations^15^.

In this study, we found several clinical risk factors of overall and physicians’ delayed diagnoses. Notably, we provided a list of misdiagnoses in ADEM (Table 2). The list contained two important messages. First, common respiratory infections, aseptic meningitis, and common gastrointestinal infections were more commonly misdiagnosed in the delayed-diagnosed group than in the early-diagnosed group (Tables 2 and 4). Therefore, one should be cautious in making these diagnoses. Meticulous investigation on whether the patients exhibit subtle signs of CNS disorders, such as encephalopathy (sleepiness), weakness, sensory deficits, and autonomic dysfunction, is necessary during follow-up. Second, the tentative diagnosis of aseptic meningitis, herpes simplex virus encephalitis, and bacterial meningitis is a forecast of ADEM. One should consider that when the infectious aetiology of intracranial inflammation is suspected, a non-infectious aetiology remains in the differential diagnosis list. In addition, we provided novel evidence that patients’ early-attendance did not affect the length of diagnostic odyssey in ADEM (Fig. 4 and Table 1). One cannot efficiently decide on the possibility of ADEM based on the length of symptoms before the patient’s initial medical visit. With these detailed data and the diagnostic odyssey plot, one can now rely on the data rather than individual physicians’ experiences and expert opinions during clinical diagnostic reasoning of ADEM.

### Study limitations

This study had three limitations. First, although ADEM is a relatively rare disease with an incidence of 0.2–0.4 per 100,000 children^18^, the small sample size in this study does not allow to obtain conclusive findings. Further validation in larger cohorts is warranted. Second, recall bias regarding patient symptoms and the frequency of misdiagnosis is possible. However, this recall bias exists in real-world clinical practices, and we believe our data can be applied to daily clinical reasoning and decision-making processes. Third, the study design limits the generalizability of the results in other areas and healthcare systems. Healthcare insurance systems, cultural differences, hospital access, and availability of investigational devices, such as magnetic resonance imaging, should be the major factors to consider in diagnostic delay studies. The addition of data through follow-up studies in other clinical settings would augment the value of our results. Nevertheless, we believe that patients with ADEM in other settings will meet similar challenges during the diagnostic processes, due to the rarity and difficulty in establishing a definitive diagnosis of ADEM.

In conclusion, a unique and long diagnostic odyssey exists before ADEM diagnosis. ADEM in children took a median of 9 days from symptom onset to diagnosis, and 83% of paediatric patients were initially misdiagnosed. A total of 61 misdiagnoses were made for 25 patients with ADEM. Several clinical risk factors associated with diagnostic delay were identified.

## Data Availability

The data are available upon reasonable request to corresponding author.

## References

[1] Pohl D (2016). Acute disseminated encephalomyelitis: Updates on an inflammatory CNS syndrome. Neurology.

[2] Cole J, Evans E, Mwangi M, Mar S (2019). Acute disseminated encephalomyelitis in children: An updated review based on current diagnostic criteria. Pediatr. Neurol..

[3] Krupp LB (2013). International Pediatric Multiple Sclerosis Study Group criteria for pediatric multiple sclerosis and immune-mediated central nervous system demyelinating disorders: Revisions to the 2007 definitions. Mult. Scler..

[4] Bisker Kassif O, Orbach R, Rimon A, Scolnik D, Glatstein M (2019). Acute disseminated encephalomyelitis in children: Clinical and MRI decision making in the emergency department. Am. J. Emerg. Med..

[5] George T (2019). Early recognition and treatment of acute disseminated encephalomyelitis in pediatrics: A case series. Pediatr. Emerg. Care.

[6] Alper G (2012). Acute disseminated encephalomyelitis. J. Child. Neurol..

[7] Yamaguchi Y (2016). A nationwide survey of pediatric acquired demyelinating syndromes in Japan. Neurology.

[8] Croft P (2015). The science of clinical practice: Disease diagnosis or patient prognosis? Evidence about "what is likely to happen" should shape clinical practice. BMC Med..

[9] Fraiman YS, Wojcik MH (2021). The influence of social determinants of health on the genetic diagnostic odyssey: Who remains undiagnosed, why, and to what effect?. Pediatr. Res..

[10] Waldrop MA (2019). Diagnostic utility of whole exome sequencing in the neuromuscular clinic. Neuropediatrics.

[11] Mefford HC (2019). The road to diagnosis: Shortening the diagnostic odyssey in epilepsy. Epilepsy. Curr..

[12] Lappe M, Lau L, Dudovitz RN, Nelson BB, Karp EA, Kuo AA (2018). The diagnostic odyssey of autism spectrum disorder. Pediatrics.

[13] Grier J, Hirano M, Karaa A, Shepard E, Thompson JLP (2018). Diagnostic odyssey of patients with mitochondrial disease: Results of a survey. Neurol. Genet..

[14] Basel D, McCarrier J (2017). Ending a diagnostic odyssey: Family education, counseling, and response to eventual diagnosis. Pediatr. Clin. North. Am..

[15] Esposito S, Di Pietro GM, Madini B, Mastrolia MV, Rigante D (2015). A spectrum of inflammation and demyelination in acute disseminated encephalomyelitis (ADEM) of children. Autoimmun. Rev..

[16] Hynson JL (2001). Clinical and neuroradiologic features of acute disseminated encephalomyelitis in children. Neurology.

[17] Pohl D, Tenembaum S (2012). Treatment of acute disseminated encephalomyelitis. Curr. Treat. Opt. Neurol..

[18] de Mol CL (2018). Incidence and outcome of acquired demyelinating syndromes in Dutch children: Update of a nationwide and prospective study. J. Neurol..

